# Conceptualizing handover strategies at change of shift in the emergency department: a grounded theory study

**DOI:** 10.1186/1472-6963-8-256

**Published:** 2008-12-16

**Authors:** Renée H Lawrence, Anne M Tomolo, Andy P Garlisi, David C Aron

**Affiliations:** 1 Center for Quality Improvement and Research 14(W), Louis Stokes Cleveland Department of Veterans Affairs Medical Center, 10701 East Boulevard, Cleveland, Ohio, 44106, USA; 2 Department of Medicine, Louis Stokes Cleveland Department of Veterans Affairs Medical Center, East Boulevard, Cleveland, Ohio, USA; 3 Department of Medicine, Case Western Reserve University School of Medicine, Cleveland, Ohio, USA; 4 University Hospital Health System, Emergency Department, Geauga, Ohio, USA

## Abstract

**Background:**

The importance and complexity of handovers is well-established. Progress for intervening in the emergency department change of shift handovers may be hampered by lack of a conceptual framework. The objectives were to gain a better understanding of strategies used for change of shift handovers in an emergency care setting and to further expand current understanding and conceptualizations.

**Methods:**

Observations, open-ended questions and interviews about handover strategies were collected at a Veteran's Health Administration Medical Center in the United States. All relevant staff in the emergency department was observed; 31 completed open-ended surveys; 10 completed in-depth interviews. The main variables of interest were strategies used for handovers at change of shift and obstacles to smooth handovers.

**Results:**

Of 21 previously identified strategies, 8 were used consistently, 4 were never used, and 9 were used occasionally. Our data support ten additional strategies. Four agent types and 6 phases of the process were identified via grounded theory analysis. Six general themes or clusters emerged covering factors that intersect to define the degree of handover smoothness.

**Conclusion:**

Including phases and agents in conceptualizations of handovers can help target interventions to improve patient safety. The conceptual model also clarifies unique handover considerations for the emergency department setting.

## Background

Clinical handovers occur when healthcare providers transfer information and primary responsibility for patient care. Clinical handovers are increasingly surfacing in discussions of patient safety, patient flow, and quality of care. [1-4] The preventable consequences of problematic handovers include delays in medical diagnosis and increases in the likelihood of adverse events in the emergency department. [5] Other consequences include higher health care costs, greater provider and patient dissatisfaction, more protracted hospital stays, and higher return visit rates. These potentially serious and overlapping consequences speak to the urgency of better understanding factors relevant to clinical handovers to maximize their effectiveness. [5,6]

There is also increasing appreciation for the role communication plays in the occurrence and understanding of medical errors [1,7], including those specific to the process of handing over care of patients. [5,8-11] Communication-based conceptual models offer a starting point of recognizing there are at least two players or agents, a sender and a receiver, and there is a medium and a message, and noise (distraction). Thus, various solutions, often revolving around minimizing communication failure, have been advanced for improving handovers. Such solutions include trying to structure the information exchanged, creating a standardized framework for the methodology of information exchange [12-16], and/or specific training on handovers and communication [17]. However, this conceptual model may also be limiting our vision. Handovers encompass more than simply communicating patient information and potential communication failures. They provide training and socialization opportunities (e.g., reinforcement of cultural practices), offer a fresh set of eyes with the potential of averting or recovering from the sequelae of adverse events, and can impact on team cohesion. [14,18,19] Moreover, it isn't clear that standardization or a 'gold standard' framework will necessarily solve the problems, and may introduce costly consequences in a process that is inherently variable. Such variability may be more than problems in the communication process. [20]

Thus, without a more complete appreciation of the process and the context within which particular handovers occur, recommended interventions and changes may inadvertently negate or eliminate the positive aspects and decrease the likelihood that meaningful changes to improve the process are achieved [20,21]. Indeed, attempts to improve handovers in emergency departments (ED) have been relatively unsuccessful [18].

Some recent ethnographic studies have contributed to understanding handovers in EDs. Patterson et al [2] generated a list of 21 coordination and communication strategies to improve handovers used in "settings with high consequences for failure," such as nuclear power plants, railroad dispatching, space shuttle mission control center, and ambulance dispatching. Behara et al [18] reported on their ethnographic observations in five emergency departments throughout the United States and Canada (all had academic affiliations) and evaluated the use of the 21 strategies, finding some strategies to be consistently used, some occasionally used and others used infrequently or not at all. While providing a useful listing of strategies, a better understanding of the processes in the ED context seems necessarily to direct development of effective interventions.

Although handovers permeate the health care system, we focused on handovers at change of shift in the ED at a large urban Veteran Affairs Medical Center (VAMC). We focused on the ED because EDs have unique considerations [18,22]. Many times the information transferred relates to patients with whom the receiving physician will most likely be interacting. As such, the oncoming physician needs to develop the coordination of care for the patients, all of whom may have very different and unrelated case scenarios and thus very different needs (e.g., all patients are not cardiac or gastrointestinal cases). This is in contrast to what occurs with most cross-coverage handovers in many other contexts (e.g., inpatient wards). We focus on the VAMC because there are important similarities with other ED settings: compressed timeframe, acute decision-intensive care contexts, heterogeneity of acuity, simultaneous management of varied acuity levels, invasive emergency procedures may need to be performed, and rapid fluctuations in patient volume and acuity levels. However, there are important differences: the focus on adult patients and the VAMC utilizes an electronic medical record (EMR) that provides almost instantaneous and comprehensive medical histories. Therefore, most patients presenting without any medical records accompanying them may not be 'unknowns.' This is in contrast to circumstances in other emergency care settings – a circumstance that is in the process of changing.

In order to develop a better understanding of handover processes in emergency medicine and provide insights that will inform interventions and redesign strategies to enhance patient care and safety [2,18,22,23], we undertook a qualitative study using observation, open-ended surveys and interviews, and included all types of ED staff. The broader goal was to conceptualize handovers in the emergency department using grounded theory and other efforts at conceptual frameworks.

## Methods

### Parameters of the setting/context

This study took place in a VAMC ED serving veterans residing in Northeastern Ohio and referrals from Veterans Affairs (VA) and non-VA nursing home care units, VA outpatient clinics, and non-VA EDs and inpatient units. The ED is part of a full-service teaching hospital with residents rotating every two weeks. It is also affiliated with 12 community-based outpatient clinics that serve veterans in northeast Ohio. The ED has a triage system that designates care to fast-track and acute areas between 8 am and 5:30 pm Monday through Friday and at all other times patients are triaged to be seen in the acute area. For physicians, Monday through Friday there are 4 changes of shifts daily (3 non-overlapping) (with the following shift start times: 8 am, 1 pm, 5:30 pm, and 6 pm). On each weekend day, physicians have 3 changes of shift (2 non-overlapping) (8 am, 8 pm and a mid-day shift). For nurses, there are 4 changes of shifts 7 days a week (3 non-overlapping) (7:30 am, 10 am, 3:30 pm and midnight). Physician Assistants (PAs) have 1 non-overlapping shift Monday through Friday that starts in the morning. Residents hand over to residents, attendings, and moonlighting attendings working nights, weekends, and holidays who serve as medical officers of the day (MOD). Attendings and MODs hand over to attendings and MODs. PAs hand over to attendings and residents. Nurses hand over to nurses. The average monthly volume during 2006 was 1969.3 patients (SD = 109.1; range = 1772–2135).

During the day shift (8 am to 6 pm) there are one to two attending physicians, three PAs, and several medical residents staffing the facility. At the 6 pm physician and PA shift changes, the MOD assumes care and responsibility for the patients. At 8 am the "night shift" physician signs out to the day shift attending physician and resident. At 8 am there generally are few or no patients in the department. Five to six interns and residents arrive at approximately 9 am. One resident joins the three PAs in the ED fast track. There are 4 to 5 interns and residents working in the acute area until 6 pm. At 1 pm the "morning shift" attending leaves, and, as needed, hands cases over to two attendings. These two "afternoon attendings" work until 6 pm when the MOD arrives and works until 8 am. The 1 pm and 6 pm handovers are the major handovers of the day for physicians.

There are 12 examination suites, 11 of which are equipped to manage critically ill patients and 1 that is a seclusion room for acute psychiatric patients. In addition there are 4 "fast track" rooms in an adjacent hallway for patients with minor injuries or illnesses.

The VAMC has an electronic medical record (EMR) and a white dry-erase board for tracking patients. The director introduced a sign-out tool for the ED that includes columns for room number, patient's name, last 4 numbers of the patient's social security number, main problem, disposition, and "to do" list.

### Design and measures

Qualitative methods were used as the best approach to clarify parameters and issues of a process that is complex and complicated by many factors, and to help identify new pieces of information about the process and participants. There were two main phases that followed an initial observation period: 1) open-ended survey and 2) in-depth interviews. This order for the methods was chosen so that information from the previous phase could be used to help inform the next phase.

The study required ethical consideration, and was reviewed and approved by the Human Studies Office (Institutional Review Board) of the VAMC Cleveland which oversees the human research protection program. Participation was voluntary and confidentiality was protected. In-depth interviews were audio recorded with permission of the interviewee (all granted permission and signed a consent form) and transcribed without any identifying information included. Once the quality of the transcription was verified, the recordings were erased.

Field observations were conducted by all three investigators at different times over a one year period and focused on physician handovers at change of shift. The objectives of the observations were to obtain a sense of the process and help formulate the study's survey and interview guide. The observers arrived fifteen to thirty minutes before the scheduled shift change and stayed for ten to fifteen minutes after the change was completed. No weekend changes of shifts were observed. One author observed over 120 handovers at the 6 pm shift change on randomly selected weekdays. Another observed a random sampling of handovers drawn from all possible change of shifts for physicians (26 hours of observation). The third investigator participated in 4 handovers per week (1 pm and 6 pm) for an extended period. Although in some ways video recording would seem ideal, it was deemed impractical given the setting: high quality recordings would be a challenge in the ED, particularly near the white board due to others working in the area, interruptions, and ambulance traffic. We also felt it unnecessary for our immediate purposes because the number of observation periods and observers most likely resulted in blending in after the first couple occasions. In addition, minimal field notes were taken during the observation period and then expanded afterwards, as is typical of ethnographic field methods.

A letter of introduction was sent informing potential participants about the survey and in-depth interviews. This was followed a week later by a copy of the open-ended survey coded for tracking purposes along with a self-addressed envelope to return the survey. Reminders and an additional copy were sent two weeks later for unreturned surveys.

The survey consisted of a cover page with questions about demographics related to the work setting and four open-ended questions. The survey was developed as a brief interview by one author and was reviewed for clarity and feasibility by the two authors who are physicians in emergency departments. The instrument was not piloted. Two versions were used, one with wording for individuals directly involved in the handover (PAs, MDs, Registered Nurses (RNs)/Licensed Practical Nurses (LPNs)) and one with wording for those not directly involved in handovers but who may be impacted (nurse assistants, health technicians, clerks). Three open-ended questions were used to identify parameters that may be currently overlooked in structured and closed-ended formats. [24,25] These questions were: (1) What strategies do you use (or have seen used) to enhance the transfer of care at change of shift? (2) What gets in the way of consistently experiencing a smooth patient handover at change of shift? (3) What suggestions do you have for improving patient handover procedures at change of shift?

After collecting and reviewing the open-ended surveys, in-depth interviews were conducted with a subset of participants. One investigator conducted interviews in a private setting removed from the ED at a time convenient to the respondent. Two versions were included, one for individuals directly involved in the handover (PAs, MDs, RNs/LPNs) and one for those not directly involved but may be impacted (nurse assistants, health technicians, clerks). The wording was changed to elicit information about what they may have observed about the process. The general interview questions and probes that were used to guide the interview were designed to elicit examples of handovers that didn't go as smoothly as expected, discussion of the benefits and risks, and strategies to deal with the risks.

### Participants

Participants for the self-administered open-ended survey included all staff members involved and potentially impacted by the handover procedures at change of shift in the ED: attendings, residents, MODs, PAs, RNs, LPNs, health technicians, and clerks. Other eligibility criteria included the ability to read and speak English. The three groups of potential participants included 51 physicians and 3 physician assistants, 18 nurses (15 RNs, 2 LPNs and 1 nurse manager), 6 clinical assistants (nursing assistants and health technicians) and 6 clerks. In-depth interviews were planned with at least one staff member from each group and would be conducted until we obtained thematic saturation, where no new ideas surface.

### Data analysis

Any identifying information was removed from all study materials and coded according to respondents' positions. Observational data was used to help develop the open-ended survey and guide the in-depth interview. Observations were also used to provide supplemental information about contextual factors and additional triangulation information for interpretation of findings.

The analytic approach chosen for analyzing the in-depth interviews was based on grounded theory [24,26,27]. The open-ended surveys were analyzed first by the lead author who created matrices to summarize responses by groups of respondents. Survey responses were summarized based on codes and categories identified in previous studies and those that emerged. The matrices were then independently reviewed for completeness and accuracy by another investigator followed by a joint discussion of the findings. The third investigator then independently reviewed the matrices. Any issues or concerns were jointly discussed. This approach ensured that a broader range of codes was identified and agreed upon.

## Results

### Participant characteristics

Thirty-one surveys (37%) were returned: 21 physicians (9 MODs; 8 attendings; 5 residents); 6 nurses (5 RNs and the Nurse Manager); 3 PAs; and 0 Clerks. Of the 31 respondents, 15 (48.4%) were female and the majority of participants was Caucasian (74%). Of the 21 physicians, internal medicine was the area of specialty for 76.2% (n = 16). Not including the residents and one respondent who did not fill in length of time they have worked in the ED, the average length of time working in the ED was 5.33 years (range: < 1 to 20 years, SD = 4.94 years).

A total of 10 interviews were conducted: 2 attendings; 1 MOD; 2 residents; 2 PAs, 1 nurse, 1 clerk and 1 health technician (the last 2 hadn't completed a survey). Forty percent of those interviewed were female and 60% were Caucasian. Excluding residents, the average length of time working in the ED for those interviewed was 4.20 years (range: < 1 to 10 years, SD = 2.97 years). Interviews averaged about 32 minutes (range: 21 to 48 minutes).

### Findings organized by open-ended items

Direct observations and interviews were used to expand understanding of issues organized around responses to the open-ended survey items.

### Handover strategies

Additional file 1 summarizes our findings and includes Patterson et al's [2] complete list of 21 strategies for direct comparison (numbered items) with the distinctions made by Behara et al [18]. The findings are based on whether the strategies were used consistently by EDs they observed, used sometimes or in some EDs, and strategies never/rarely used by EDs they observed. In the table, we use all capital letters to flag the additional/new strategies and/or elaborations to Patterson et al's list based on the current study.

Our findings lend further support to strategies identified by Patterson et al [2] and reported by Behara et al [18] as strategies used consistently and strategies used sometimes or in some institutions. However, we found 5 of the 9 strategies classified by Behara et al as never used or rarely used [18], were used or some variant of the strategy was used with more frequency in our setting. Those 5 items are included in a separate column and the additional information in parentheses gives the application of the strategy in our setting as provided by our respondents and confirmed by observation. Thus, for example, while no complete "read-back to ensure information was received" occurred, our data revealed that fact checking is a strategy used by the incoming along with the outgoing printing and handing pending transfer information to the incoming. Consistent with Behara et al [18], we did not observe, nor did our respondents report, any of the strategies in the fifth column. Additional strategies we found are presented in the last column.

In analyzing the issues and themes, a more comprehensive understanding of the process necessitated a discussion of the primary agent or agents linked to the strategies [23]. "Agent" represents the main focus of and/or player (primary actor) in the strategy, such as the initiator of an action, decision maker, or source of support for the strategy, and based on previous findings and the current data, includes the following categories: outgoing, incoming, other staff, and cultural/environmental factors.

For example, strategy #16, where the incoming receives paperwork that includes handwritten annotations, is classified in our matrix under preparatory phase and aligned with the outgoing party as the agent because the key time for the strategy was *prior *to the handover and done by the outgoing to hand to the incoming. Thus, for some strategies there is one primary agent. The actual handover phase is where the formal exchange takes place and both agents are primary (dyad is primary).

Both direct observation and analysis of responses identified two other types of important agents: other staff and cultural/environmental factors. While some discussions have recognized the incoming and outgoing [2,18,23], few have explicitly conceptualized other agents. By "other staff" it is meant that other parties not directly involved in the often like-like (e.g. MD-MD) handover are somehow involved (e.g., other staff actually provide information or additional updates) or informed by the handover (e.g., overhear others' updates which therefore provides some additional information and/or opportunity for correcting misinformation). By cultural/environmental agent we mean considerations or factors that impact on the various phases of the handover that are part of or shape the ED culture, such as staffing issues, shift definitions, and general protocol or expectations for the handover that involve more than the primary agents.

Moreover, to fully appreciate the strategies, it was clear from our observations and analyses that it is important to identify phases of a handover to fully understand the processes surrounding the handover, consistent with other recent approaches [23]. Accordingly, we differentiated the following phases: 1) Anticipatory, 2) Preparatory, 3) Handover, 4) Immediate Post-Handover Phase and the 5) Post-Handover Phase that continues forward and whose length is a function of the time it takes to resolve the cases handed over. The strategies presented in Additional file 1 are mapped out according to agent and phase of the handover process in Additional file 2.

For example, one type of strategy relates to reducing the number of handovers and engaging in timely admissions. Such activities suggest an anticipatory phase where the outgoing party is actively anticipating the upcoming handover and engages in behaviors to reduce the number of patients that have to be handed over (e.g., push for transfer to floors so the case is out the door before handover) and/or ensure that some handovers are completed or brought to closure so that they don't have to be handed over (e.g., recheck to see if lab results are entered/back, make a call to check on labs to determine final disposition), or there is minimal work needed if they do have to be handed over (e.g., disposition determined and follow-up steps taken for disposition decision so that minimal work is needed by the receiver of the case). Thus, issues relevant to enhancing the handover clearly begin before the actual handover.

Similarly, the preparatory (prep) phase is a separate phase that happens just prior to the actual handover phase (where the two agents meet and exchange information), and reflects activities to prepare for the handover such as updating information, writing a summary, preparing handwritten annotations to provide to the incoming agent. The next phase is the actual handover and includes numerous strategies to enhance the transfer of care, such as efforts to limit interruptions, giving the incoming information about care plans and contingency plans, update information during the handover, see patients or make walking rounds. The immediate post-handover phase is that window of time right after the exchange. Strategies related to this include the outgoing staying to complete or bring cases to closure as much as possible so that the incoming can attend to new cases or other cases in the midst of the work-up and/or treatment. For the incoming, the immediate post-phase strategies include seeing patients, particularly those that are less stable or complicated, and reassessing and refining plans. The post-handover phase reflects that time when the environment is adjusting to the new staff taking over the shift. The cycle of phases then repeats itself as the next change of shift approaches.

### Obstacles to smooth patient handovers and suggestions to improve the process

**Figure 1:**
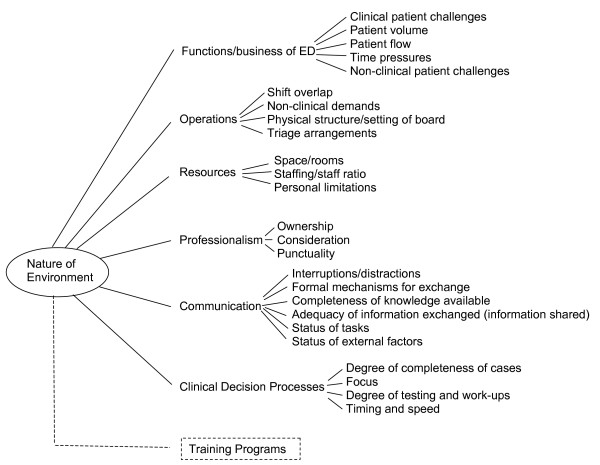
**Mapping of themes and categories related to handovers in the emergency department**.

Analyses of the questions about obstacles and suggestions to improve the process revealed similar themes and categories, with the suggestion often addressing the obstacle. As such, Additional file 3 summarizes the combined findings. We have included group-specific references for the actual responses or items (i.e., type of staff mentioning the item) by phases in the handover process. The first two columns provide the categories and themes identified. Figure 1 provides a visual summary of the themes and categories identified by respondents.

Specifically, six general themes or clusters emerged covering the gamut of factors that intersect to define the degree of smoothness and patient safety involved in a particular handover. It is important at the outset to recognize the interconnectedness of the categories and themes. There was one non-general theme that related to training of residents.

Functions or the business of the ED theme encompasses aspects that are part and parcel of an ED. The categories that emerged included clinical patient challenges (e.g., high acuity patients, multiple high acuity patients presenting at the same time), patient volume (e.g., the number of patients seen, the hectic nature of the setting), patient flow (patients moved to make room, patients length of stay in the ED is too long), time pressures (the environment by nature has time constraints related to doing the work), and non-clinical patient challenges (e.g., patients moved to chairs and they become hostile and hard to handle creating additional work for staff).

"Operations" relate to aspects and decisions made about how to structure the ED to meet those aspects that, by definition, are part of the setting. The categories under this theme are shift overlap (e.g., whether or not there is overlap and who focuses on what patients, schedules demand that some people leave on time and without overlap that person isn't available to answer questions), non-clinical demands (e.g., in-service training and staff meetings), physical structure/setting of the board (e.g., crowding in the area and the chaotic nature of the setting around the board), and triage arrangements (e.g., have one triage location, improve triage process).

"Resources" relate to categories that were mentioned relevant to physical space/rooms (e.g., a high acuity patient comes in but there's no plan of where to put that patient) and personnel, including staffing ratio and personal limitations (fatigue) that impact on the handover process.

"Professionalism" also emerged with three general categories: ownership (e.g., out-going is viewed by on-coming as responsible for new physician orders which creates different perspectives about who is responsible); consideration (e.g., reluctance to accept handover or inconsideration); and punctuality (e.g., arrives on time and starts on time).

"Communication" relates to various factors that impact on the process and nature of information exchange. The categories include interruptions/distractions that occur during the time of the actual handover that make it challenging and reduce the smoothness of the process (e.g., phone calls, pages going off), formal communication mechanisms for the sign-out process (e.g., some had recommendations for creating a more formal and consistent procedure, such as walking sign-outs or rounding at the beginning of the shift, or elimination of an approach, such as dictating notes that would have to be reviewed and transcribed rather than entering them in electronic fields), completeness of knowledge available (e.g., gaps in patient knowledge or familiarity create challenges, such as patients on gurneys or chairs being less well-known), adequacy of information exchanged (e.g., the amount of information shared, including as new requests or changes are added or made), status of tasks (e.g., provide more specifics about time test ordered, notified and done), and external factors (e.g., clarify directions for transfers).

Another theme or cluster relates to aspects of "clinical decision processes" that impact on the smoothness of the handover. We identified four categories within this theme: degree of closure or completeness of the cases (e.g., patients are handed over with too many loose ends, decisions aren't made quickly regarding disposition); focus of attention (e.g., focused more on new patients rather than being as concerned about cases that are being handed over); degree of testing and work-ups (e.g., excessive testing creating work that is perceived as unnecessary for the purposes and functions of an ED); and timing and speed (e.g., number of requests coming in at once and wanting requests addressed quickly).

## Discussion

The importance of understanding handovers is well-recognized. Patient handovers in an emergency department present unique challenges and obstacles. Patients have an undifferentiated presentation and are generally of higher acuity than those on general medical wards. Clinical status changes can be measured in timeframes of seconds to minutes for emergency department patients with unstable medical conditions. Previous attempts to organize handover coordination and communication strategies, although providing an important foundation, are conceptually limiting. We have expanded previous efforts by differentiating two critical factors (timeframe and agents) consistent with recent human factors approaches [23]. Expanding previous work by explicitly including and expanding the timeframe of the handover process and incorporating different phases not only increases the understanding of this process but also facilitates the development of more focused interventions to improve the handover process [23]. While our anticipatory, preparatory, handover and immediate post-handover phases map out to phases suggested by Grusenmeyer and used by others [23], we found that appreciating strategies that may begin in a pre-handover phase is helpful, as was thinking about the post-handover phase. Outlining these phases provides a better understanding of the handover (a limited process) that occurs within a larger context, and permits the addition of flexibility in the approach to a handover depending upon the pace of the day in the unpredictable work environment of the ED. For example, during a more chaotic shift the providers may provide the best handover by simply focusing upon the brief preparatory phase and preparing to extend their time to the post-handover phase to finish their cases that are near a final disposition rather than hand the case(s) over. However, in shifts with a more consistent pace of work the providers may anticipate the handover as they consider the extent of patient evaluations and testing, and the context of other departments as they prepare for the handover. Updates on the context may include status of ED ancillary staff and capacity of inpatient services.

Explicit consideration of the obvious agents also provides a more comprehensive approach to organizing strategies and interventions [23]. Our findings necessitate explicitly acknowledging various agents and addressing considerations that are often missing from most handover models: other staff and the culture/environment. If ED providers now consider these other agents in the handover process they may consistently incorporate other staff in the handover or consider converting the actual handover into a communication huddle that includes other key personnel such as the charge nurse and lead clerk in addition to the outgoing and incoming providers. Expanding the handover to incorporate these other voices permits discussion of cultural and environment factors that may be unapparent to the providers. Expanding this communication also permits additional staff to participate in the pre-handover and post-handover phases, which may relieve some responsibilities from the providers, for example, updates on status of radiology and lab testing and inpatient unit capacity.

As emphasized by Behara et al. [18], the ED has unique considerations that distinguish it from other services to which it is intimately linked. One key consideration is the type of handover that is involved in change of shifts: the content and needs related to the handover quite literally vary from patient to patient. Behara and colleagues summarized these issues by noting that standardization is very low in emergency handovers, patient flow is unpredictable and varies, and the likelihood of the oncoming provider having to interact with the case that is handed over is very high. This is in contrast to inpatient wards where the handover is often more similar than different. As such, the strategies and ultimately the interventions to improve the handover process require appreciating the differences and the similarities.

**Figure 2:**
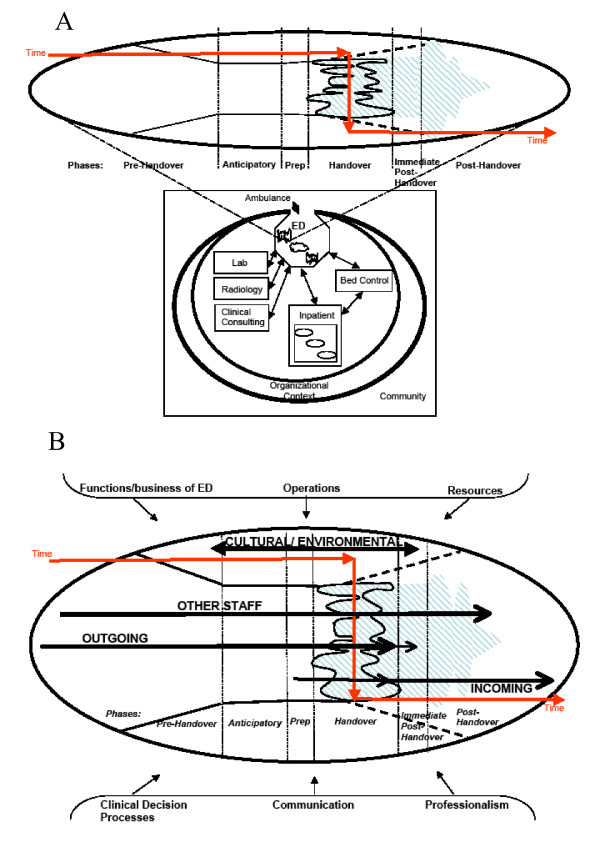
**Schematics of conceptual domains and issues related to understanding handovers in emergency departments**. ED – Emergency Department. → – Core communication and interdependence patterns that impact on ED processes.

Figures 2a and 2b provide a schematic to summarize the various conceptual issues that emerged. The bottom half of Figure 2a is designed to help position the ED within the larger context. In this portion the key interdependent relationships between departments within the hospital are represented and connected by bidirectional arrows that represent the flow in communication and impact. These departments and the ED exist within a larger context of the macro-organization which also has influence over the operation and communication within the hospital. The "open door" relationship with the community is represented with bidirectional arrows as ambulances bring patients to the ED and may also take them to other facilities. This "door" to the community may be shut in cases when the ED is on diversion due to a variety of potential overflow situations within the hospital.

The top half of figure 2a provides an enlarged version of the handover process, integrating the phases or timeframe of the process. The direction of the flow across time is represented by a series of linked arrows that change direction during the actual handover phase. In general the work of the handover decreases or becomes more focused in the phases preceding the actual handover. The shape of the actual handover phase in the top half of the model is shaped to reflect the variability in time and issues involved with each case that is handed over (which is in contrast to inpatient wards where the handovers are represented by a consistent shape in Figure 2a). In some cases the handover continues into the oncoming's shift with varying degrees of demand on the outgoing and incoming providers, including becoming more demanding before resolving, as may be the case with a patient with acute changes in medical status around the time of the handover.

Figure 2b focuses on the ED and overlays all the conceptual issues and themes. Agents are included and the model provides a useful reference point for understanding any handover that occurs within the ED setting or involves the ED setting. By overlaying the themes, the model provides a means of appreciating the interrelatedness of the various conceptual dimensions. As such, communication is one part of the interplay and interventions that focus solely on communication, may lose sight of other factors and/or the interrelationship among factors. Thus, a structured handover tool may help with some issues but will not help with other factors, many of which may be unique to the ED setting or uniquely played out in this setting. And, in fact, some of the most viable interventions to improve the actual handover phase, may end up not being directly part of that phase but rather, for example, relate to how the themes, including communication, play out in the pre-handover phase.

One challenge to smooth handovers that was identified related to excessive testing and the reference to the 'internal medicine focus,' with the suggestion being "don't do the million dollar work-up on everyone." This issue cuts across all phases of the handover process and suggests opportunities related to training since our ED is staffed by mostly internal medicine trained physicians who are also training residents in the context of the ED. Thus, one recommendation would be to take advantage of the presence of residents. Specifically, this suggests that a training program may be developed to educate trainees to the unique aspects related to the nature of providing care in the ED. This training may bridge the gap in knowledge of trainees and remind the other providers that the focus of the ED is upon timely identification, management, and disposition of patients with acute and sub-acute problems. As trainees return to the ED during their residency the principles of this training can be reinforced by having the returning residents contribute to the teaching of their peers who are on their first rotation in the ED.

This training might also include other critical staff, such as the nurses and physician assistants, giving them a more visible role in the training process including the critical nature of the handover in care and the preparation for a successful handover. This presents an opportunity to highlight the importance of communication between nursing and physicians that was described by some respondents as inadequate in our study, particularly increasing the awareness of the "plan of care" for the nursing staff.

Other considerations to improve the handover relate to emphasizing the finding that the handover exchange is critically embedded in a process and a context, which suggests recommendations that while not directly altering the actual handover, may improve it. In particular, the anticipatory and preparatory phases could easily be lost or truncated in a chaotic environment such as the ED, resulting in compromised or lost strategies related to these phases. Technology or environmental supports for these phases of the process to prevent losing track of time and suddenly realizing it is time to handover cases might be helpful to avoid incomplete information at the time of the handover or a prolonged handover. For example, if an electronic tracking system is available, it is possible to change the color of the screen two hours before change of shift and then having a blinking screen 40 minutes before hand. Also, other environmental cues, such as reminder or warning bells, might be helpful with or without electronic tracking systems. These cues could help to support those strategies directly related to those phases and at the same time, since the intervention informs all parties (e.g., clerks as well as providers), help with other challenges such as the overall flow of patients (e.g., fewer patients on gurneys who are not well known) and reinforcing timeliness of activities (e.g., remind the clerks to call bed control again about the status of inpatient beds).

On a related note, appreciating the embedded nature of the ED and the phases of the handover process results in recommendations sensitive to the whole context. Focusing on challenges that impact on all phases, as summarized in Additional file 3, suggests the importance of patient flow within and across shifts. Recommendations to address many of the patient flow concerns necessitate resolving broader organizational or system issues such as improving updates to and from bed control, and transfer mechanisms.

In addition, as the providers and other ED staff work to adopt some of the strategies described for the anticipatory and preparatory phases of the handover, and to acknowledge the influence of the larger health care system, there may be an opportunity to develop a tool or checklist to assist with the management of the actual handover. That is, as other broader issues get addressed, the inherent variability in the handover moment may be lessened by smoothing other interrelated components across the phases, making a tool that helps to structure the exchange potentially more useful. At the same time, the tool and its implementation will need to be flexible given the very nature of the frequently changing pace and composition of work in the ED. It may be helpful to consider the communication challenges described in this study when developing this tool. Also, with the inclusion of electronic tracking system that integrates with the electronic medical record, there may be opportunities to develop a tool that incorporates the results of patient testing. Additionally, an electronic tracking system that includes a handover tool permits updating by providers and clerical and nursing staff, which again may facilitate communication of patients' plan of care.

There are limitations to our study that should be acknowledged and placed in context. Our objective was to identify strategies, opportunities and challenges related to handovers in the urgent care/emergency department. The response rate for the open-ended surveys was 37%. While we considered this a good response rate for an open-ended survey in a group that works in a fast pace environment, was experiencing staff turnover, and was dealing with demanding and unpredictable clinical cases, the analysis did not include written input from some of the participants in the handover process. Therefore, although we included field observations, we may still be missing strategies, challenges, and opportunities despite having achieved saturation. We feel this is unlikely given the multiple methods and the overlap with findings from other larger studies. Similarly, we did not design the study to determine how effective or important strategies were. However, by investigating barriers to smooth handovers, we believe we have identified issues relevant to ensuring that handover strategies are effective and important. Another limitation may be the lack of an electronic or computerized tracking system that has replaced white boards in some ED settings. While the electronic tracking boards render some strategies less relevant because they permit updating patient status and diagnostic testing, the ability to create and modify clinical information and decision-making in real time remains problematic with these electronic systems. It is worth noting that since completing this study, our ED put in a computerized tracking system.

In addition, we are limited to one setting that potentially has some unique circumstances, including that it is a teaching environment, a VAMC with only an adult patient population, electronic medical records, and an emergency department staffed with primarily non-emergency medicine trained physicians. Nevertheless, staffing by non-emergency medicine trained physicians is a common arrangement both in the VA and elsewhere. A follow-up study that includes emergency departments of varying organizational and contextual parameters would help further our understanding of the factors that impact on successful and less successful handovers. Finally, our focus was on only one type of handover: change of shift. However, the findings have relevance for most transitions in terms of appreciating both the similarities and differences.

Despite these limitations, the findings expand important frameworks that are evolving to better understand transitions of care in emergency care settings. These frameworks will provide the foundation to implement successful patient and provider safety improvements in this unique setting. [2,18,20,23] Thus, although generalizability remains to be addressed in future research endeavors, our findings may have direct relevance for implementation efforts in other settings.

## Conclusion

The importance and complexity of handovers, the commonly occurring situation in which both information and primary responsibility for a patient are transferred, is well-established. This study of clinical handovers identified limitations in the current approaches to conceptualization. Using a grounded theory approach, we developed a more comprehensive conceptual framework that will facilitate improvement of this problematic transition in care. Based on assessment of strategies, obstacles to smooth handovers, and suggestions for improving handovers, we identified relevant themes, phases, and core agents of the handover process as key conceptual components. The conceptual model also clarifies unique handover considerations for the emergency department setting.

## Competing interests

The authors declare that they have no competing interests.

## Authors' contributions

RHL conceived and designed the study, survey and interview protocol, conducted interviews and analyses, developed the conceptual themes and model, and drafted and revised the manuscript. AMT conceived and designed the study and survey, helped with analysis and development of the model and manuscript. APG participated in the design of the study, analysis and interpretation of the data, and provided feedback on the conceptual model and revisions. DCA provided feedback about the design of the study and helped with revising of the manuscript. Authors are listed in order of contribution, and all read and approved the final manuscript.

## Pre-publication history

The pre-publication history for this paper can be accessed here:



## Supplementary Material

Additional file 1 **Table 1**. Strategies reported or observed to enhance transfer of care at change of shift organized by primary agent of the strategy Click here for file

Additional file 2 **Table 2**. Conceptualizing strategies reported or observed to enhance transfer of care at change of shift organized by agent and phase in the Emergency Department handover process Click here for file

Additional file 3 **Table 3**. Themes and categories of challenges to smooth handovers and suggestions for improving handovers Click here for file
