# Supplementary material for: Conceptualizing handover strategies at change of shift in the emergency department: a grounded theory study
Source: BMC Health Serv Res. 2008 Dec 16;8:256. doi: 10.1186/1472-6963-8-256 (PMC2640383; doi:10.1186/1472-6963-8-256)
Supplement: Additional file 1 — Table 1. Strategies reported or observed to enhance transfer of care at change of shift organized by primary agent of the strategy [file 1472-6963-8-256-S1.doc]

**Table 1. Strategies reported or observed to enhance transfer of care at change of shift organized by primary agent of the strategy**

| **AGENT** | **Strategies Used Consistently in ED Based on Behara et al and Current Study** | **Strategies Used Occasionally in ED Based on Behara et al and Current Study** | **Strategies Behara et al Listed as Never/Rarely Used *but* Used/Reported in Present Study** | **Strategies Never or Rarely Used/Reported in ED Based on Behara et al and Current Study** | ***ADDITIONAL* Strategies Occasionally Observed/Reported in Current Study** |
| --- | --- | --- | --- | --- | --- |
| **OUTGOING** | Outgoing has knowledge of previous shift activities - 14  Delay transfer of responsibility when concerned about status/stability of process - 21  Limit initiation of operator actions during update - 5  Include outgoing team’s stance toward changes to plans and contingency plans -6  **(PROVIDE FLAGS/HEADS UP ABOUT CASES/ TO DO ITEMS)**  Update information in the same order every time - 10 | Oversees incoming’s work after update – 20 | Writes summary before handover - 8  Incoming receives paperwork that includes handwritten annotations -16  **(UPDATE INFORMATION/ TRACKING SHEET)**  Receives primary access to the most up-to-date information -15  **(DISTRIBUTES PREPARATORY TRANSFER INFORMATION)** | Intermittent monitoring of system status while off/on call - 13 | **REDUCE NUMBER OF HANDOVERS BY PLANNING AHEAD & RESOLVING ISSUES**  **PUSH FOR TIMELINESS OF ADMISSIONS**  **UPDATE BOARD**  **WRITE/ENTER BULLET POINTS ON BOARD**  **COMPLETE (BRING TO CLOSURE) AS MUCH AS POSSIBLE ABOUT EACH CASE** |
| **OUTGOING 🡨🡪**  **INCOMING** | Face-to-face verbal update with interactive questioning - 1  Topics initiated by both – 4 |  |  |  | **SEE PATIENT - USE WALKING ROUNDS**  **NEGOTIATE**  **DIVISION OF**  **LABOR**  **UPDATE BOARD DURING HANDOVER** |
| **INCOMING** | Assesses current status – 9 |  | Read-back to ensure that information was accurately received -7  **(in the partial form of FACT CHECKING)** | Reviews automatically captured changes to sensor-derived data before update – 12  Scans historical data before update – 11 | **ASK NOTE BE PLACED BY OUTGOING**  **WRITE INFORMATION DURING HANDOVER**  **SEE PATIENTS: RE-ASSESS AND REFINE CARE PLAN** |

**Table 1 continued. Strategies reported or observed to enhance transfer of care at change of shift organized by primary agent of the strategy**

| **AGENT** | **Strategies Used Consistently in ED Based on Behara et al and Current Study** | **Strategies Used Occasionally in ED Based on Behara et al and Current Study** | **Strategies Behara et al Listed as Never/Rarely Used *but* Used/Reported in Present Study** | **Strategies Never or Rarely Used/Reported in ED Based on Behara et al and Current Study** | ***ADDITIONAL* Strategies Occasionally Observed/Reported in Current Study** |
| --- | --- | --- | --- | --- | --- |
| **OTHER STAFF** |  | Additional update from practitioners other than the one being replaced - 2  Overhear others’ updates – 19 |  |  |  |
| **CULTURAL/**  **ENVIRONMENTAL** |  | Unambiguous transfer of responsibility -17 | Make it clear to others at a glance which personnel are responsible for which duties at a particular time – 18  **(HAVE ROOM ASSIGNMENTS & CHANGES POSTED)** | Limit interruptions during update/handover - 3 | **GET ADEQUATE STAFFING & STAGGERED SHIFTS**  **ALLOCATE STAFFING FOR PATIENT OVERFLOW IN ED** |

Note. Numbered items correspond to Patterson et al. (2004) listing of 21 handover strategies for all settings: space shuttle mission control, nuclear power, railroad dispatching, and ambulance dispatching.
