# Supplementary material for: Conceptualizing handover strategies at change of shift in the emergency department: a grounded theory study
Source: BMC Health Serv Res. 2008 Dec 16;8:256. doi: 10.1186/1472-6963-8-256 (PMC2640383; doi:10.1186/1472-6963-8-256)
Supplement: Additional file 2 — Table 2. Conceptualizing strategies reported or observed to enhance transfer of care at change of shift organized by agent and phase in the Emergency Department handover process [file 1472-6963-8-256-S2.doc]

**Table 2. Conceptualizing strategies reported or observed to enhance transfer of care at change of shift organized by agent and phase in the Emergency Department handover process**

| **AGENT** | **ANTICIPATORY PHASE** | **PREPATORY (PREP) PHASE** | **HANDOVER PHASE** | **IMMEDIATE POST-HANDOVER PHASE** | **POST-HANDOVER PHASE** | |
| --- | --- | --- | --- | --- | --- | --- |
| **OUTGOING** | Outgoing has knowledge of previous shift activities -14 | | |  |  | |
|  | Delay the transfer of responsibility when concerned about status/stability of process -21 | Writes summary before handover - 8; Incoming receives paperwork that includes handwritten annotations -16; **(UPDATE INFO/ TRACKING SHEET)** | Limit initiation of operator actions during update -5 |  |  | |
|  | **REDUCE (BRING TO CLOSURE) NUMBER OF CASES THAT HAVE TO BE HANDED OVER; TIMELINESS OF ADMISSIONS** | **UPDATE BOARD; WRITE/ENTER BULLET POINTS ON BOARD** | Include outgoing team’s stance toward changes to plans and contingency plans -6  **(PROVIDE FLAGS/HEADS UP ABOUT CASES; TO DO ITEMS)** | Oversees incoming’s work after update – 20 |  | |
|  |  |  | Update information in the same order every time -10 | **COMPLETE (BRING TO CLOSURE) AS MUCH AS POSSIBLE** |  | |
|  |  |  | Receives primary access to the most up-to-date information -15 **(DISTRIBUTES PREPATORY TRANSFER INFORMATION**) |  |  | |
| ***OUTGOING***  ***🡨🡪***  ***INCOMING*** |  |  | Face-to-face verbal update with interactive questioning -1 |  |  | |
|  |  |  | Topics initiated by both - 4 |  |  | |
|  |  |  | **SEE PATIENT/DO WALKING ROUNDS** |  |  | |
|  |  |  | **NEGOTIATE DIVISION OF LABOR** | |  | |
|  |  |  | **UPDATE BOARD DURING HANDOVER** |  |  | |
| **INCOMING** |  | Reviews automatically captured changes to sensor-derived data before update – 12 | Read-back to ensure that information was accurately received -7 ;  **(in the partial form of FACT CHECKING)** |  | |  |
|  |  |  | **ASK NOTE BE PLACED BY OUTGOING** |  | |  |
|  |  |  | **WRITE INFO DURING HANDOVER** |  | |  |

**Table 2 continued. Conceptualizing strategies reported or observed to enhance transfer of care at change of shift organized by agent and phase in the Emergency Department handover process**

| **AGENT** | **ANTICIPATORY PHASE** | **PREPATORY (PREP) PHASE** | **HANDOVER PHASE** | **IMMEDIATE POST-HANDOVER PHASE** | **POST-HANDOVER PHASE** | |
| --- | --- | --- | --- | --- | --- | --- |
|  |  | Assesses current status – 9 | | **SEE PATIENTS: RE-ASSESS AND REFINE PLAN** | |  |
|  |  | Scans historical data before update – 11 |  |  | |  |
| **OTHER STAFF** |  |  | Additional update from practitioners other than the one being replaced -2 |  | | Intermittent monitoring of system status while off/ on call -13 |
|  |  |  | Overhear others’ updates – 19 |  | |  |
| **CULTURAL /ENVIRONMENTAL** |  |  | Limit interruptions during update/handover -3 | Unambiguous transfer of responsibility -17 | |  |
|  | **ADEQUATE STAFFING & STAGGERED SHIFTS** | | | | | |
|  | **ALLOCATE STAFFING FOR PATIENT OVERFLOW IN ED** | | | | | |
|  |  |  | Make it clear to others at a glance which personnel are responsible for which duties at a particular time – 18, **(CLEAR ROOM ASSIGNMENTS)** | | |  |

Note. Numbered items correspond to Patterson et al. (2004) listing of 21 handover strategies for all settings: space shuttle mission control, nuclear power, railroad dispatching, and ambulance dispatching. Bolded, capitalized items do not have numbers because they are expansions or additions based on the current study.
