# Supplementary material for: Conceptualizing handover strategies at change of shift in the emergency department: a grounded theory study
Source: BMC Health Serv Res. 2008 Dec 16;8:256. doi: 10.1186/1472-6963-8-256 (PMC2640383; doi:10.1186/1472-6963-8-256)
Supplement: Additional file 3 — Table 3. Themes and categories of challenges to smooth handovers and suggestions for improving handovers [file 1472-6963-8-256-S3.doc]

**Table 3. Themes and categories of challenges to smooth handovers and suggestions for improving handovers**

| **THEME** | **Category** | **Anticipatory** | **Prep** | **Handover** | **Immediate-Post** |
| --- | --- | --- | --- | --- | --- |
| **FUNCTIONS/**  **BUSINESS OF ED** | **Clinical patient challenges** |  |  | Potential cardiac and high acuity patients (RN)  High acuity in at time of handover (AT***)*** |  |
|  | **Patient volume** |  | Rush of seeing patients and preparing handover (R) | Busy (R ); high census/overload (RN; AT); high volume of patients to sign out (AT)  Large influx of patients at peak times/Too many patients (RN, PA); The more patients, the less smooth the handover is (MOD)  Chaos of high velocity days (AT, R, RN )  Chaos (AT, R ); Busy (MOD; R)  Hectic nature of other things; Business in ED (AT; MOD)  New patient arrivals (MOD); |  |
|  | **Patient flow** | Patients moved to chair to get new patient in room (RN) |  | Patient movement from rooms to hall (RN); Long stays (RN); |  |
|  | **Time pressures** |  |  | Time constraints (RN) |  |
|  | **Non-clinical patient challenges** | Patients moved to chair who become hostile – time consuming to manage these patients (RN) |  |  |  |
|  |  |  |  |  |  |
| **OPERATIONS** | **Shift overlap** |  |  | No overlap of shifts (RN, MOD); Suggest have 1 physician focus on old patients and 1 focus on new (AT); Time constraints – need to leave on time so rushed, can’t ask questions (AT; RN; MOD) |  |
|  | **Non-clinical demands** |  |  | Delay due to in-service and staff meetings (RN) |  |
|  | **Physical structure/setting of the board** |  |  | Crowding; lack of room in area; (AT) Chaotic environment around board (AT); Residents get in way when busy with patients (MOD); Lack of room on board (MOD) |  |
|  | **Triage arrangements** |  |  | One triage location for all incoming (RN); Improve triage (PA) |  |

**Table 3 continued. Themes and categories of challenges to smooth handovers and suggestions for improving handovers**

| **THEME** | **Category** | **Anticipatory** | **Prep** | **Handover** | **Immediate-Post** |
| --- | --- | --- | --- | --- | --- |
|  |  |  |  |  |  |
| **RESOURCES** | **Space/rooms** |  |  | Potential cardiac/high acuity patients with no plan where to put them (MOD; AT) |  |
|  | **Staffing/ Staff ratio** | Need a charge nurse who doesn’t see patients each shift (RN; AT) |  | Too many MDs transfer care over to one MOD (R; PA; MOD)  Not enough staff (RN)  More nurses (RN; PA;MD)  Staff constraints (RN) |  |
|  | **Personal limitations** |  |  | Fatigue (AT) |  |
|  |  |  |  |  |  |
| **PROFESSIONALISM** | **Ownership** |  |  |  | Out-going viewed by on-coming as responsible for new physician orders during handover period (RN) |
|  | **Consideration** |  |  | Annoyed or reluctant to accept handover (PA); inconsideration (rare) (R) |  |
|  | **Punctuality** |  |  | Need to start on time; attending coming late (RN; AT) |  |
|  |  |  |  |  |  |
| **COMMUNICATION** | **Interruptions/**  **distractions** |  |  | Nurses/questions; phone calls; pages during transfer (AT), large influx of patients (PA);  Physician crowding around board; vocal interruptions (PA) |  |
|  | **Formal communica-tion mechanisms for sign-out process** |  | Notes dictated for signed out patient is a problem because of delay in obtaining/getting dictated notes (MOD); Writing notes interferes (R; RN) | No formal mechanism and one is needed (AT; RN; MOD; PA)  Need group handover (AT);  Need walking sign-out (AT)  Suggest more complicated patients go last (PA) | Attending and charge nurse SHOULD round at beginning of shift (RN) |
|  | **Completeness of knowledge available** |  | Previous nurse unaware of potential risks due to lack of information shared (RN) | Problems with patients on gurneys/chairs since they are not well known (AT) |  |
|  | **Adequacy of information exchanged (information shared)** | Trying to transfer and MD says can’t, I ordered another test/med - unaware until then (RN) | Lack of communication from physician about patient’s previous health history (RN) | Insufficient communication between MD & RN (RN); Update not conveyed but put on board (RN); nurses not aware of plan of care/new tests (RN) | Oncoming walks in and told by patient the doctor hasn’t told me anything (RN) |
|  | **Status of tasks** |  | Update orders sheet so specific with time test ordered, notified, done (RN MOD) |  |  |
|  | **Status of external factors** |  | Clarify directions regarding transfers from other hospitals (MOD); bed availability (MOD) |  |  |

**Table 3 continued. Themes and categories of challenges to smooth handovers and suggestions for improving handovers**

| **THEME** | **Category** | **Anticipatory** | **Prep** | **Handover** | **Immediate-Post** |
| --- | --- | --- | --- | --- | --- |
|  |  |  |  |  |  |
| **CLINICAL DECISION PROCESSES** | **Degree of wrap up of cases** |  | Decisions not made quickly regarding disposition (RN); no idea for disposition (MOD); Poor follow-up of patient results prior to handover (MOD) | Multiple patients with unclear or inaccurate info (MOD); too many loose ends (MOD) |  |
|  | **Focus** |  |  | Problem when oncoming is more concerned about new patients than handover cases in midst of workup (R ) |  |
|  | **Degree of testing and work-ups** |  | Excessive testing and Internal Medicine focus; don’t do million $ work-up on everyone (RN; MOD) |  |  |
|  | **Timing and speed** |  | Problems with sudden decision and want everything done at once even though patient been there a while (RN); Unable to prioritize due to too many requests at same time (RN) | Demands related to new physician orders (RN);  Need moratorium on new orders (unless emergency) RN |  |
|  |  |  |  |  |  |
| **TRAINING** | **Staffing/staff ratio** |  |  | Too many trainees (RN) so crowded; Distractions with precepting the trainees (AT) |  |
|  | **Completeness of knowledge** |  |  | Both resident and attending switch and no one knows patient well (AT) |  |

Note: Group-specific references are provided in parentheses: AT (attendings) (n=8), MOD = nightshift medical officer of day (n=9), R (resident) (n=5); RN (n=6), PA (n=3)
